# The hard life of an octopus embryo is seen through gene expression, energy metabolism, and its ability to neutralize radical oxygen species

**DOI:** 10.1038/s41598-024-67335-9

**Published:** 2024-07-17

**Authors:** Sadot Ramos-Rodríguez, Karen Ortega-Ramírez, Luisa Méndez-Can, Clara Galindo-Sánchez, Pavel Galindo-Torres, Claudia Ventura-López, Maite Mascaro´, Claudia Caamal-Monsreal, Gabriela Rodríguez, Fernando Díaz, Carlos Rosas

**Affiliations:** 1 Laboratorio de Genómica Funcional, Departamento de Biotecnología Marina, Centro de Investigación Científica y de Educación Superior de Ensenada (CICESE), CP 22860 Ensenada, BC México; 2 Unidad Multidisciplinaria de Docencia e Investigación (UMDI), Facultad de Ciencias UNAM, CP 97355 Sisal Yucatán, México; 3 Unidad de Química en Sisal, Facultad de Química UNAM, CP 97355 Sisal Yucatán, México

**Keywords:** Biochemistry, Genetics, Physiology

## Abstract

The reproductive process in Octopus maya was analyzed to establish the amount of reactive oxygen species that the embryos inherit from females, during yolk synthesis. At the same time, respiratory metabolism, ROS production, and the expression of some genes of the antioxidant system were monitored to understand the ability of embryos to neutralize maternal ROS and those produced during development. The results indicate that carbonylated proteins and peroxidized lipids (LPO) were transferred from females to the embryos, presumably derived from the metabolic processes carried out during yolk synthesis in the ovary. Along with ROS, females also transferred to embryos glutathione (GSH), a key element of the antioxidant defense system, thus facilitating the neutralization of inherited ROS and those produced during development. Embryos are capable of neutralizing ROS thanks to the early expression of genes such as catalase (CAT) and superoxide dismutase (SOD), which give rise to the synthesis of enzymes when the circulatory system is activated. Also, it was observed that the levels of the routine metabolic rate of embryos are almost as high as those of the maximum activity metabolism, which leads, on the one hand, to the elevated production of ROS and suggests that, at this stage of the life cycle in octopuses, energy production is maximum and is physically limited by the biological properties inherent to the structure of embryonic life (oxygen transfer through the chorion, gill surface, pumping capacity, etc.). Due to its role in regulating vascularization, a high expression of HIf-1A during organogenesis suggests that circulatory system development has begun in this phase of embryo development. The results indicate that the routine metabolic rate and the ability of *O. maya* embryos to neutralize the ROS are probably the maximum possible. Under such circumstances, embryos cannot generate more energy to combat the free radicals produced by their metabolism, even when environmental factors such as high temperatures or contaminants could demand excess energy.

## Introduction

Global warming is changing the ocean environment and affecting marine life. Coastal zones are particularly vulnerable because they are exposed to temperature and oxygen changes, sea level rise, typhoons, hurricanes, temperature increments, hypoxic zones, pH changes, and reduction in fisheries^1^. Global warming is also affecting marine life. Coastal areas are particularly vulnerable because temperature directly influences cephalopod life cycle^2–4^,which is the main factor that modulates embryogenesis duration. Furthermore, thermal condition especially affects embryonic development and growth, yolk absorption and hatching rate^5,6^. In several cephalopod species, embryogenesis duration can be shortened when egg incubation is performed at high temperatures within the species optimal temperature range^5,7,8^. Temperatures at thermal range extremes generate morphological anomalies and death of embryos, low hatching success, and deficiency in the use of the internal yolk in newborn offspring, causing low growth rates and short survival times^4,9–11^. However, the life history of females may also be important for embryo development. Before spawning, female octopuses acquire the energy for egg production directly from food, which is vital for embryos and offspring because their nutritional condition influence the morphological and biochemical characteristics of the next generation^12–14^. Recent studies in *O. maya* demonstrated that thermally stressed females produce embryos with higher metabolic rates than animals from non-stressed females^15,16^. In O. maya was demonstrated that thermally stressed females produce embryos with higher metabolic rates than animals from non-stressed females15, 16. High levels of oxidant damage (OD) and ANTIOX enzyme reduction were also observed in embryos from stressed females, which explain the limited ability of offspring to neutralize excess OD^11^. In *O. mimus* embryos from females without an additional stressor, a recent study reported low levels of antioxidant/detoxifying (ANTIOX) enzymes (ANTIOX) and a high load of reactive oxygen species (ROS) in the first development (blastulation and organogenesis) stages^17^. Additionally, once the heart and circulatory system activities are initiated, the ANTIOX enzyme activation reduces ROS levels, indicating that the embryos are prepared to buffer the ROS transferred from female once organogenesis has been completed. As a result of Olivares et al., the present study proposes the hypothesis that ROS inherited from females are produced in females due to the metabolic processes that occur in the ovary during the latter part of the yolk synthesis^17^. This hypothesis also considers that due to an adaptation, the embryo genome should have all the genetic machinery to produce ANTIOX enzymes that allow reducing the maternal and own ROS produced during embryonic life. Since reproductive and developmental processes accompany the dynamic changes in metabolism and energy consumption, byproducts—as ROS species—are also inevitably generated on an extraordinary scale during the physiological oxygen consumption process^18^. Until now, to our knowledge, no study has proven if octopus females transfer OD to octopus embryos. During embryo development, animals should multiply cells, synthesize organs, and absorb yolk for growth. From the embryos perspective, these processes consume the energy stored in the yolk before completing their development. Thus, one way of knowing how much energy is still available could be based on evaluating the aerobic scope (AS). According to Fry^19,20^, the AS is the difference between the maximum (MMR) and standard metabolisms (SMR), which represent the energy surplus beyond the energy needed to maintain life and directed to grow and maintain homeostasis. Although until now, no information on the cephalopod embryo AS has been available, a study made on chicken observed that during the embryo development, the AS was absent^21^. These results were obtained by measuring the chicken oxygen consumption in normothermia and hyperthermia; the AS was calculated as the difference between both values. The results of the study previously mentioned showed that the embryo routine aerobic pathways were close to the maximal rates obtained in hyperthermia. This result indicated mitochondria and all the metabolic pathways of chickens in routine conditions are at their highest operative metabolism because, routinely, all the energy is directed to growth. In other words, chicken embryos do not need a higher metabolism than the routine, consequently, they have no aerobic scope^21^. Is there the same in octopus embryos? If it is, then the production of ROS during development should follow the metabolic activity, being the embryo obligated to neutralize both ROS transferred from females and those produced by itself^17^. Previous results in *O. maya* embryos have shown increment in the routine metabolic rate per embryo, reaching their higher values at the end of the development stage^7,11^ suggesting that as observed in chicken, in *O. maya* the AS could be reduced because is highly probable that all the energy is directed to embryo development. In non-athletic animals or embryos, AS can be measured as thermal metabolic scope (TMS^22^). This method allows calculating the surplus energy flux proportion left after the basal maintenance costs of the organism are reached^23,24^thus reflecting the energy embryos can invest in somatic production and other fitness-related functions^23,25^. In practice, TMS is calculated as the difference between the high metabolic rate (HMR) obtained when animals are exposed to a temperature that positively stimulates metabolism and provokes a low metabolic rate (LMR)^22,26,27^. According to Caamal-Monsreal *et al.*, 30 °C stimulates *O. maya* metabolic rate when embryos are maintained for one hour or less without damage^7^. On the other hand, laboratory results (data unpublished) indicate that exposure at 11 °C for one hour allows obtaining a low metabolic rate, facilitating AS evaluation of embryos maintained in laboratory conditions, following the TMS method^22^. Although the protein enzymatic activity and gene expression happen at different levels within the cell where the synthesized information is found, they can provide an approximation of how different cellular and metabolic processes work. For some enzymes, a correlation exists between the relative gene expression and enzyme activity. Although they are not always correlated in the case of antioxidant enzymes, they can help determine whether the cell is under oxidative stress^28,29^. In this sense, to our knowledge, the present study is the first of a series of experiments using *O. maya* as the model to evaluate if female thermal stress is a key factor in OD transfer, and if the reasons why these octopus species are sensitive to warming scenarios can be explained even though they are inhabiting a tropical zone. Consequently, the aim is directed to answer the following questions: (i) How does the embryo AS function during development? (ii) If ROS is produced in female *O. maya* ovaria, how do they transfer to eggs and embryos? (iii How and when do *O. maya* embryos reduce the ROS transferred from females and produced during the embryo's respiratory metabolism? (iv) When is the ANTIOX enzyme genetic machinery turned on in embryos allowing them to be prepared to neutralize the OD before hatch.

## Results

### Eggs and embryos wet weight

**Figure 1:**
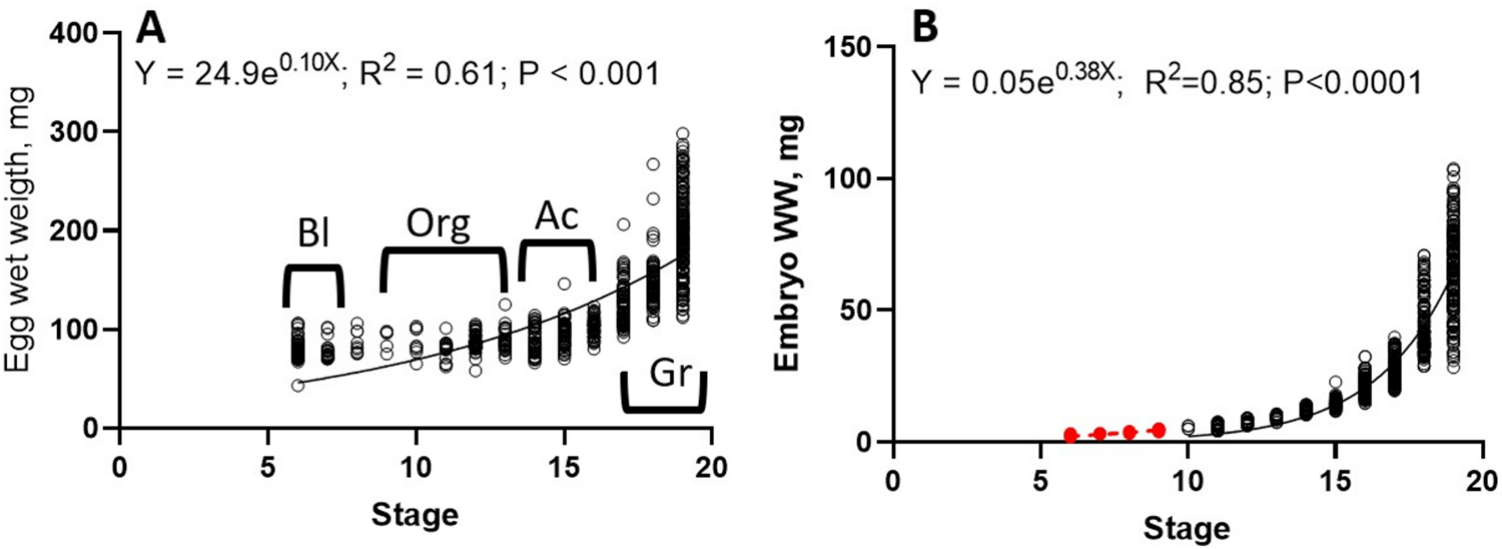
Changes in living weight of eggs (**A**) embryos; (**B**) *O. maya* acclimated at 24 °C (Raw data). The embryo development stage was defined as blastulation (Bl) (stages 6 and 7) when an eyespot appears, and usually the embryo is placed in the distal position of the gonopore after the first reversion; organogenesis (Org) was characterized by the presence of internal organs in the mantle (stages 8 to 14), and before that, the heart starts its activity; Activation (Ac) was defined when the embryo heart starts its activation (stages 15 to 16); Growth (Gr) (stages 17 to 19) is defined between activation and the end of embryo development after hatch. The dark line shows the mean wet weight (mg) of embryos measured in this study. Red points indicate the embryo estimated wet weight using equation (**B**). n = 1265 embryos, all maintained at 24 °C and collected during 5 years.

During embryo development the weight of 725 eggs and embryos was recorded and the relationship between the stage and weight was constructed with both eggs and embryos (Fig. 1A). Small changes on wet weight (ww) were recorded in eggs between blastulation and at the end of the organogenesis stages with values oscillating from 69 to 80 mg ww. When the embryo was activated starting the heart functions and circulatory system, a change in weight was recorded reflecting the embryo growth phase (Fig. 1A). It should be noted that the variability of egg weight enhances with embryo development. At the end of the embryo phase, we registered egg weight recorded from 112 to 298 mg (Fig. 1A). Changes in *O. maya* embryo wet weight were recorded along their development (Fig. 1B), which was performed once the animal was evident into the egg (stage X) and until stage XIX. With those data, a second exponential curve was calculated (Fig. 1B). Values from 4.2 to 103 mg of ww were recorded with a higher variation at the end of the embryo development stage. Using the equation obtained, the embryo wet weight were calculated during the end of blastulation and at the beginning of organogenesis (stages VI to IX) (Fig. 1B). Values from 1.77 to 4.89 were estimated for embryos in those initial stages (Fig. 1B).

### The oxygen consumption of embryos

**Figure 2:**
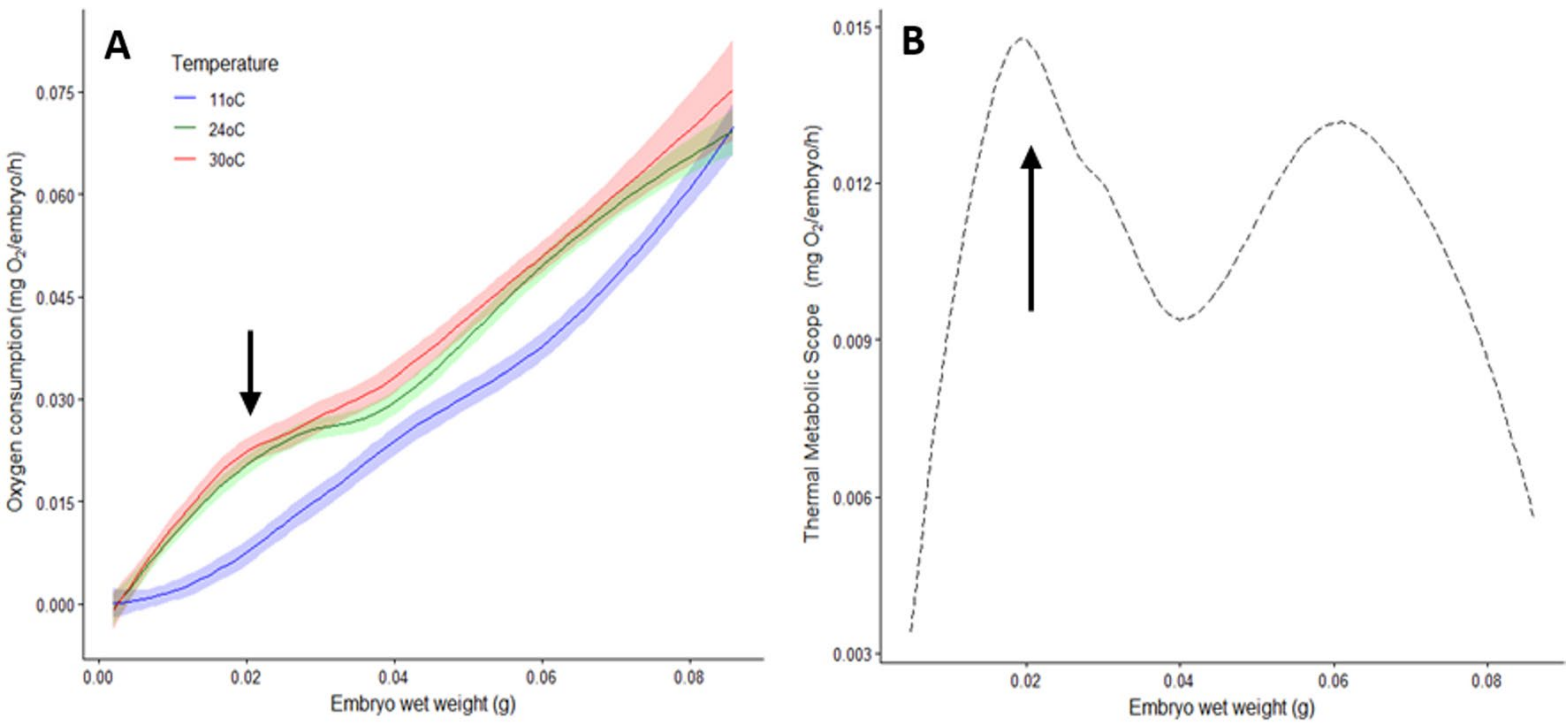
Embryo respiratory metabolism (mg O_2_ h^−1^ embryo^*−*1^) as a function of embryo wet weight (g) in embryos acclimated at 24 °C and acutely exposed to 11 (n = 354) and 30 °C (n = 362) (**A**). Thermal metabolic scope (TMS); (**B**) was calculated as the difference between predicted oxygen consumption values of embryos exposed at 11 and 30 °C. Arrows indicate the weight of embryos when the heart and the circulatory system start their function, defining the activation phase and the beginning of embryo growth.

As expected, oxygen consumption increased with the weight of the embryos and following embryo development (Fig. 2). Oxygen consumption of the embryos exposed at 11 °C was lower than that obtained in those exposed at 24 and 30 °C. The relationship between MO_2_ and embryo weight (Loess analysis) showed that the MO_2_ of the embryos exposed at 11 °C had lower increments with the weight following a power curve during organogenesis and changing to be lineal when the activation phase onwards was reached (Fig. 2A). In contrast, the embryos measured at 24 and 30 °C showed similar curves with a high increment of the MO_2_ from 0.088 to 0.095 g embryo wet weight, just during the activation phase (Fig. 2A). After the activation phase, a plateau was recorded in the embryos measured at 24 and 30 °C, with weights higher than 0.015 g wet weight. With those data, the thermal metabolic scope (TMS) was calculated (Fig. 2B). As observed, the maximum TMS was obtained during the activation phase, just when the embryos’ heart and circulatory system start at the beginning of the growth phase. Another peak was recorded in embryos during growth when they had around 60–70 mg wet weight. Before hatch a reduction on TMS was observed with lower values at the end of embryo development (Fig. 2B).

### Antioxidant defense mechanisms (ANTIOX) and oxidant damage (OD)

**Figure 3:**
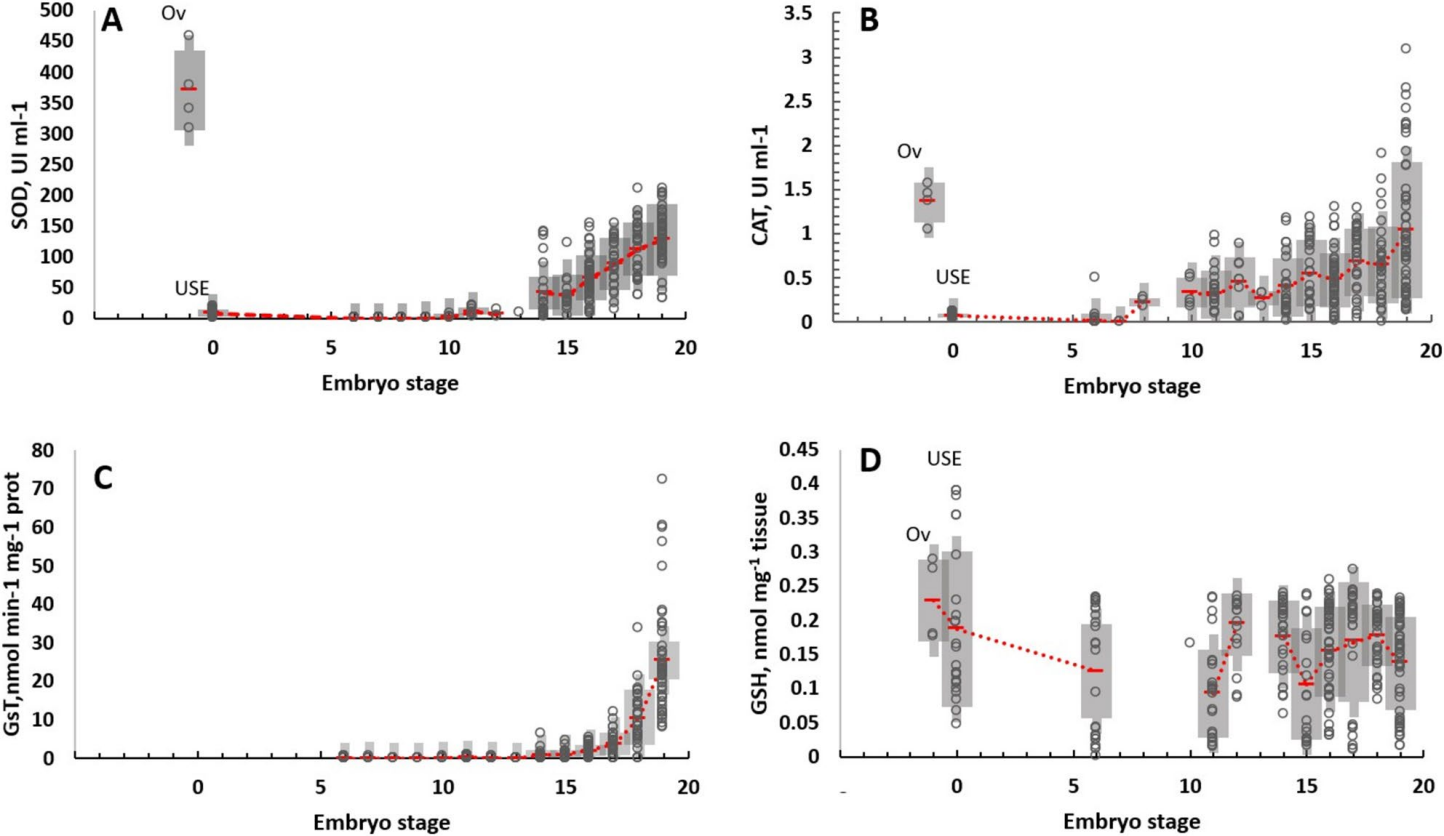
Antioxidant defense mechanisms measured in ovaria (OV), in un-spawned eggs (USE) of *O. maya* females (n = 5) and in their eggs (unspawned eggs; n = 55), embryos in different stages of development and incubated at 24 °C (GST, n = 216; (CAT, n = 303), (SOD, n = 275), GSH, n = 236). Red dashed lines connecting mean ± SD values (grey shadows). Raw data as empty points. Superoxide dismutase (SOD; **A**); Catalase (CAT; **B**); Glutathione-s-transferase (GsT; **C**); Total glutathione (GSH; **D**).

**Figure 4:**
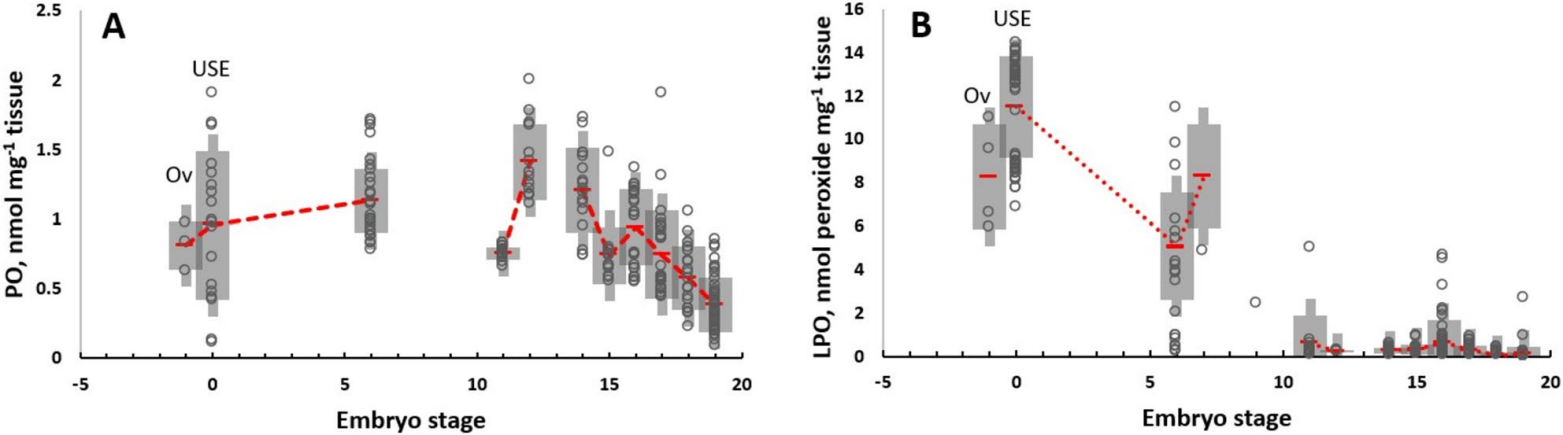
Oxidant damage registered in ovaria (OV) and in un-spawned eggs (USE) of *O. maya* females and their embryos incubated at 24 °C. Red dashed lines connecting mean + SD values (gray shadows). Raw data as empty points. Lipo-peroxidation (**A**; LPO, n = 194), Protein oxidised (PO, n = 197; **B**).

Antioxidant defense mechanisms (ANTIOX) enzymes (SOD and CAT) were active in octopus females (Fig. 3) and coupled to high levels of PO and LPO in ovaria (Fig. 4). It is interesting to note that ANTIOX in ovaria was 350 and 150 higher in ovaria than in the un-spawning eggs (USE), SOD, and CAT, respectively (Fig. 3). During the embryo development, ANTIOX activity was still lower until stage X–XI, where a slight increment of SOD and CAT activity was observed. An increment in the activity of three enzymes measured (SOD, CAT, and GsT) was recorded when the embryos reached stage XIV onwards (Fig. 3A–C). Total glutathione (GSH) had values that oscillated between 0.1 to 0.23 nmol mg^*−*1^ tissue without significant changes during the embryo development (Fig. 3D). Oxidant damage (PO, and LPO) in the ovaria of the females was high in USE and in the first stages of embryos, until stages X–XII, where a continuous reduction was observed. Values lower than 0.5 nmol mg^*−*1^ tissue were observed at stage XIX, just before hatch (Fig. 4A,B).

### Gene expression analysis during embryo development

**Figure 5:**
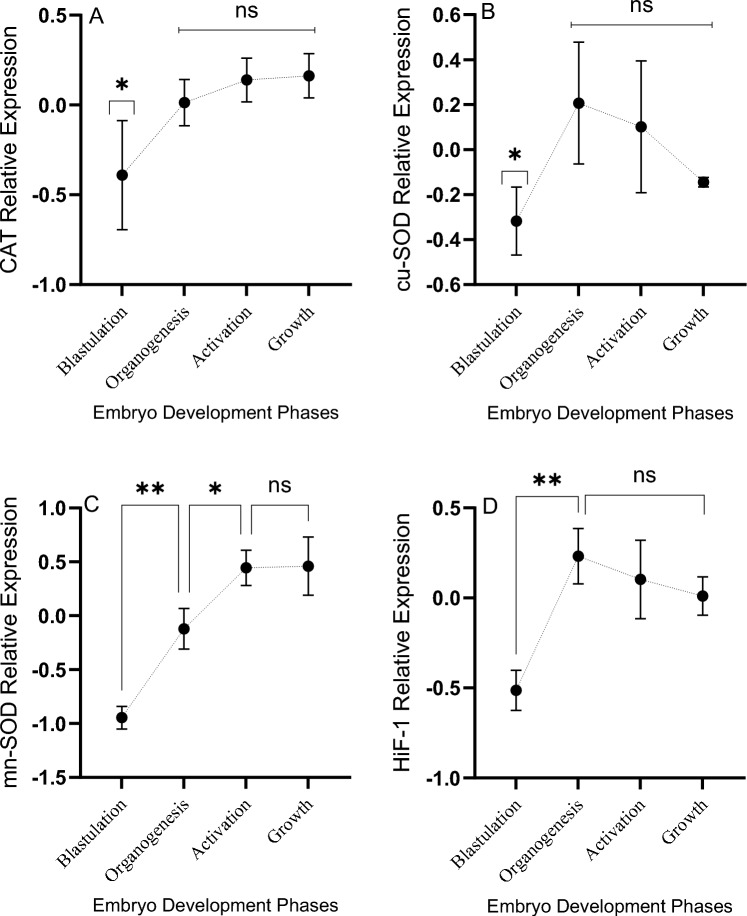
Changes of the relative expression of the catalase (CAT), cupper-superoxide dismutase (cu-SOD), manganese-superoxide dismutase (mn-SOD) and hypoxia-inducible factor 1-alpha (HiF-1) of de *Octopus maya* embryos development. Values as mean ± SD of the relative expression expressed as Ln. Statistical differences at 0.02 (*) and 0.003 (**) levels. Blastulation: stages VI–VIII; Organogenesis: stages IX–XIII; Activation: stages XIV–XVI; Growth: stages VII–XIX.

The present study used TufM and GPAM for nomalizing the quantitative expression of the target genes, since they were the most stable genes found by the stability analysis. The catalase (CAT) gene was found to be expressed in all the embryonic stages; However, differences between the evaluated stages were not significant (*P* = 0.117); the mean expression shows a tendency to increase through embryo development (Fig 5) with the lowest expression in the blastulation stage. A similar expression pattern was observed for the manganese superoxide dismutase gene (MnSOD), where significant differences between stages were observed (*P* = 0.0017) being lower at blastulation and higher at the growth stage (Fig. 5). For superoxide dismutase (SOD) (Fig 5) and HIF1A genes, an inverted expression was observed when compared to MnSOD and CAT, that is, the expression of both genes decreased with the development of the embryos, reaching a higher expression at organogenesis and decreasing in the following stages. For HIF1A, the expression at the blastulation stage was significantly lower (*P* = 0.018) than for the rest of the evaluated stages.

## Discussion

Naef^30^ initiated three periods of embryonic development characterized as (i) formation of blastoderm, (ii) gastrulation with the establishment of endoderm and mesoderm, and (iii) organogenesis that ends when the embryo hatches^31^. In the present study, identifying the phase of blastoderm formation phase was difficult in *O. maya* embryos because the eggs were sampled until the female presumably finished spawning, seven days after the blastoderm formation had finished. Consequently, identifying the end of blastulation (stage VI–VII) was possible in a few days before the start of organogenesis and beyond until hatching^30^.In this sense, *O. maya* embryos were characterized in four general stages of development, (1) blastulation (2) organogenesis (3) activation and (4) growth.

To our knowledge, the exponential relationship between egg wet weight vs stage and embryo wet weight vs stage have been obtained for the first time. Interestingly, while the entire egg does not change within the stage until activation, more acute changes on embryos wet weight were recorded in the stage. The results indicate that as organogenesis develops, the changes in embryo weight are probably compensated by the small amounts of yolk consumed and the absorbed water, generating the idea that the egg weight does not change between blastulation (stages 6–8) to activation phase (stages 14–16). However, when the yolk and peri-vitelline fluid are removed and the weight of the embryo is measured, the real weight is obtained showing—as expected—a well-defined exponential curve. Thus, if the embryo entire egg is used to calculate oxygen consumption, very likely, an overestimation of metabolic rate should be obtained. Previous results obtained in *O. vulgaris*^32^, and *O. mimus*^8^ overestimated the oxygen consumption of embryos because the entire egg was used to express metabolic data by weight unit. The present study evaluated the standard metabolism of embryos using the TIMR method, where a low temperature (11 °C) was used to depress the routine metabolism, as a proxy of standard metabolism and also a high temperature (30 °C) was used to obtain the maximum metabolic rate. With those values, the thermal metabolic scope was calculated as the difference between maximum and low respiratory metabolism^22^. The results obtained showed that routine metabolism is close to the maximum metabolic rate, suggesting that embryo metabolism cannot increase higher than they have during their development when maintained in optimal temperature range (24–26 °C). The results indicate that routine metabolic rate of *O. maya* embryos is probably the maxima possible, probably due to limitations imposed by the limited number of mitochondria, oxygen exchange rate between seawater and perivitelline liquid, and the possible role of the chorion as an oxygen-barrier. Other studies should be performed to understand (1) how mitochondria concentration changes with embryo development; (2) how oxygen flows through the chorion and temperature modulates the oxygen dissolved in perivitelline liquid of this octopus species. The results obtained in the present study show that the thermal metabolic scope (TMS) peaks when embryos reach the state of activation and organogenesis ends. Although a new peak was observed during the growth phase after a low TMS value was recorded, the downregulated processes could have been involved to limit the embryo energy demands during the last part of the growing phase.

A hypothesis could help to explain these results Marthy et al.^33^ described a natural tranquillizer in the perivitelline fluid of loliginid squid, which prevents premature hatching. Although that substance has not been identified in octopod species yet, possibly similar molecules could be acting in octopus embryos, provoking a reduction of the metabolic rate before hatch. Another hypothesis could be related with the dissolved oxygen availability at the end of the growth phase. Before hatch, an increment on standard oxygen consumption was observed indicating that the energy demands to satisfy the basal metabolism of embryos are at their maximum level. In contrast, a reduction in the routine metabolic rate and maximum oxygen consumption of embryos were observed also at the end of the embryo development, indicating that basal, routine and maximum metabolic rates have close values at this stage. In such circumstances, a reduction in dissolved oxygen in the perivitelline fluid at the end of the growth phase could be also expected due to the high oxygen demands of the embryo that cannot be satisfied by the oxygen in the perivitelline fluid limited by the chorion surface where they are enclosed. Without enough oxygen the reduction in the maximum metabolic rate observed could explain the reduction in the maximum metabolic rate at the end of development, resulting in a reduction in the thermal metabolic scope. Although the oxygen concentration in perivitelline fluid is not known, evidence indicates that brooding octopus females stimulate the embryos to hatch, expelling water directly to the eggs, probably in an attempt to maintain higher oxygen dissolved in the environment (To review see Villanueva and Normal et al., 2008)^34^. Although a relationship between the mechanical stimulation provided by brooding female on the egg mass and oxygen concentration in fluid perivitelline in *O. maya* embryos is not known, possibly females could be promoting a high oxygen dissolved level to prevent hypoxia and protect the embryos before hatch.

**Figure 6:**
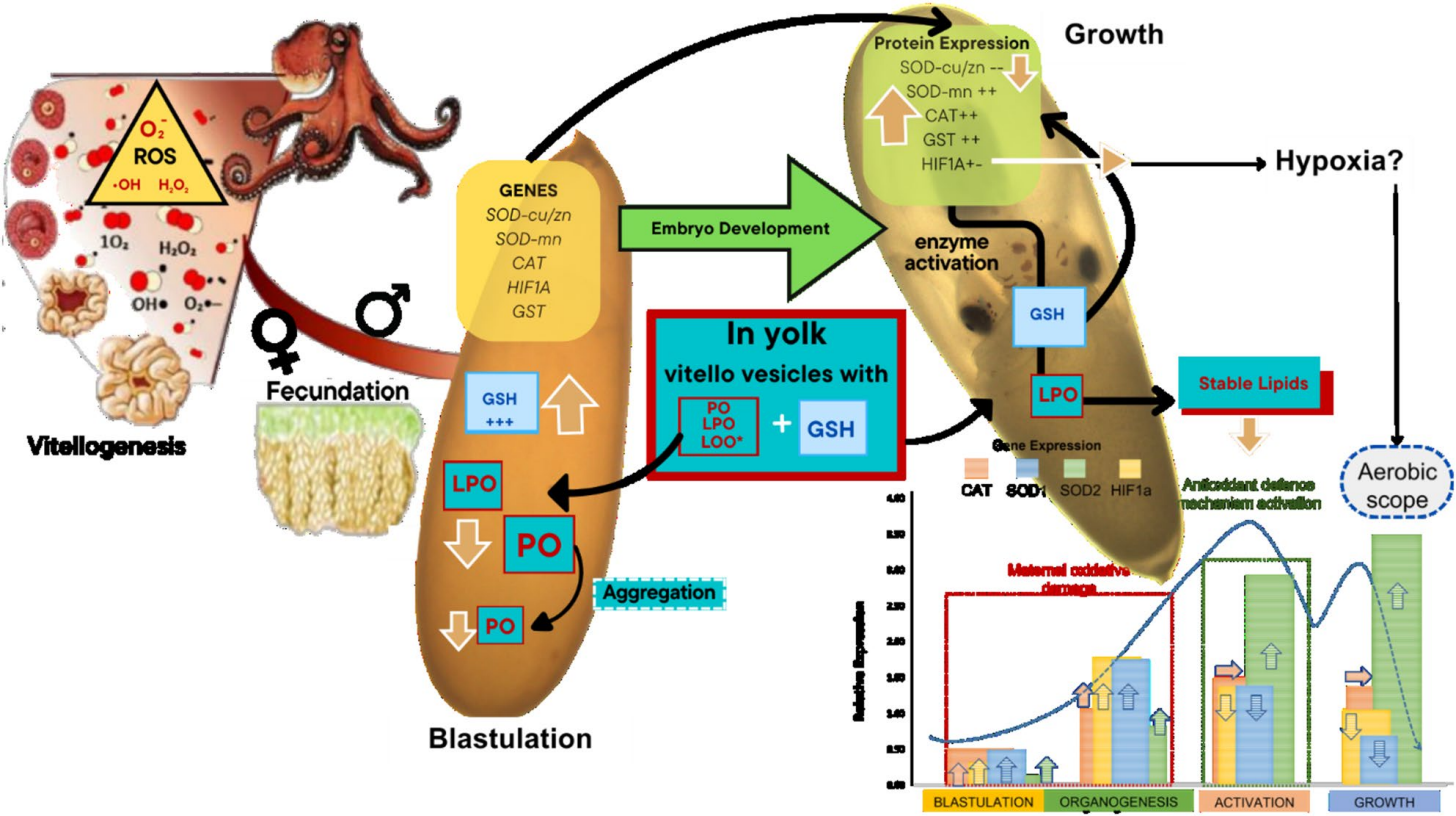
Based on the results obtained in the present study is proposed a scheme to show how hard is the octopus embryo life. ROS generated in the female are partly transferred to the egg during the metabolic processes of ovarian maturation. In the yolk, in addition to the ROS (identified as lipoperoxidation: LPO; protein oxidized: PO), the female placed GSH molecules into the yolk, presumably using yolk vesicles^58^, directly giving the embryo one of the key molecules of the glutathione system, which will allow the neutralization of LPO and other ROS placed by the female in the yolk. The results suggest that this GSH package facilitates the activation of the glutathione system which should increase during embryo development, following the increment of metabolic rate and the ANTIOX mechanisms. The genetic machinery is activated in the embryo in the blastulation phase, provoking an increment in mn-SOD, cu-SOD, and CAT expression recorded during organogenesis, indicating an increase in ANTIOX defense components and their activities in that phase of development. At the same time, HIF-1 also registered an increment in expression, suggesting increments in lipoprotein and carbohydrate metabolism. While CAT and mn-SOD reach their maximum expression in the activation phase and are still stable during the embryo's growth, at the same time, a reduction in the expression of cu-SOD was recorded, suggesting that the cytoplasmic cu-SOD role is finished at the end of the growth phase. The HIF-1 expression was still almost constant during embryo development, indicating its role as a regulator of glucose transporter 1 (GLUT-1) and the most glycolytic enzymes linked to anaerobic metabolism. The relative stability of HIF-1 suggests that embryos experience some level of hypoxia during development, which could be related to the reduction in thermal metabolic scope observed towards the end of embryo development.

The present study evaluated changes on some enzymes involved in antioxidant defense mechanism in the ovarium, unspawned eggs and along the development of *O. maya* embryos. Additionally, LPO and PO were evaluated in the same tissues to know how the relationship between the female and embryos is during yolk synthesis and embryo development (Fig. 4). In females, it is noteworthy that elevated LPO and PO values were observed in the ovarium (Ov), un-spawned eggs (USE), and embryo early developmental stages. Several studies have proposed a relationship between reproduction and oxidative stress^35^.Reproduction is an energetically costly process for females, increasing resource requirements, metabolism, and potentially ROS production^35–37^. It has been observed in *Octopus mimus* ROS produced during metabolic processes has been observed to occur during ovarian maturation and in part transferred to the egg, causing a maternal ROS load to the embryo^17^. This result coincides with those observed now in the present study indicating that during yolk formation, the female transfers an amount of peroxidized lipids to un-spawned eggs that must be neutralized during embryo development (Fig. 6).

In the present study, a considerable increase of SOD, CAT, and GsT was observed from the activation stage onwards, when the circulatory system is activated in stages XIV–XVI. That pattern was also observed in *O. mimus* embryos^38^, indicating that antioxidant defense mechanisms are activated as a response against the embryo ROS production, increasing energy production to support the embryo growth phase. Catalase is one of the ROS detoxifying enzymes that can be found in the early embryonic development stages, which its activity remains practically stable until hatching in several species^39–41^. CAT and SOD are considered the first line of defense against ROS because they directly neutralize the oxygen singlets. In consequence, the embryos could need synthesizing both enzymes at the beginning of their development to maintain the balance between prooxidant-antioxidant homeostasis^42^, which explains the expression levels detected from the blastulation stage with an increase in organogenesis remaining stable until hatching. Superoxide dismutase (SOD) is a ubiquitous family of enzymes that efficiently catalyzes superoxide dismutase anion. To date, three superoxide dismutases (SODs) have been biochemically and molecularly characterized, whose structure and function are highly conserved for many species^43^.

SOD1 or CuZn-SOD is a copper- zinc-containing homodimer found almost exclusively in intracellular cytoplasmic spaces^44^ and SOD2 or Mn-SOD exists in the cell as a tetramer and is initially synthesized with a leader peptide, which directs this manganese-containing enzyme exclusively to the mitochondrial spaces^45,46^. The present study shows that SOD1 expression is current from the first development stage, while the activity of the enzyme starts during the organogenesis stage. Other studies observed that the specific activities of these enzymes follow a characteristic increase with development and growth. However, embryonic enzyme activities do not necessarily correlate precisely with mRNA levels. Abramov and Wells^39^ and El-Hage and Singh^47^ have suggested that mRNA specific to these genes may accumulate and not be immediately translated. Therefore, the increase in the expression of all the genes evaluated in the present study could represent a preparation of the embryo towards the activation stage, which metabolically is a critical one due to the beginning of the circulatory system functioning and embryo growth^8,11,17^. Although the HIF1A gene is a crucial mediator in response to hypoxic conditions, it is also induced by ROS^48^. Furthermore, HIF1A is essential for embryonic vascularization^49,50^ as it regulates the production of vascular endothelial growth factor A (VEGF-A)^51^. Therefore, at this stage, a high HIF1A expression is reflecting that the circulatory system development has begun. In *O. maya* embryos, vascularization and antioxidant response coincided during the organogenesis stage, indicating that the circulatory system and ROS neutralization are coordinated. It is essential to consider that ROS transferred from female to embryos could alter these processes, mainly when the parents are exposed to thermal stress^38^. In addition, while the circulatory system remains incomplete, O_2_ supply to developing embryonic cells could be diffusion limited, which could lead to a lack of energy. Although further studies are required to test this hypothesis, possibly in such circumstances, anaerobic metabolism could be activated to help maintain energy levels. HIF-1A controls almost all the aspects of anaerobic metabolism by regulating genes, such as glucose transporter 1 (GLUT-1) and most glycolytic enzymess^52,53^, suggesting that HIF1A high expression could be indicating that oxygen dissolve in the perivitelline liquid could be limited, provoking a reduction on high metabolism when the hatch process is closed. When the follicular cells start the process of yolk synthesis, peroxidized lipids and oxidized proteins are placed into the eggs probably as a by-product of the follicular cell metabolism.

The results obtained in the present study showed that high levels of LPO and PO were recorded in the ovarian and the unspawned eggs. Moreover, high levels of GSH in unspawned eggs suggest that this antioxidant agent was packed as part of the molecules that are placed in the yolk to be used during embryo development. The tripeptide GSH (γ-glutamyl-cysteinyl-glycine) is considered one of the essential antioxidant agents. It protects cell membranes against lipid peroxidation. GSH is present in cells in millimolar concentrations and functions as a redox buffer to maintain the overall cellular redox state. However, it also acts as a cofactor for the enzymes GRx, GPx, and GST, which are present to catalyze the Glutathione oxide-reduction cycle using NADPH within the glucose metabolism pathway. ^54,55^. Although how GSH is placed in octopus embryos is still unknown, recently vitello vesicles were recorded and isolated in chicken egg yolk filled with GSH^56^. When the vesicles were supplemented in pork embryo culture medium, an increment of GSH content and reduction of ROS generation in pork embryos maintained in vitro were recorded suggesting that a charge of GSH is placed in the egg to help the embryo neutralize the ROS produced in females during the yolk synthesis ^56^. Although until now there is no evidence of vitello vesicles in the octopus yolk, in another species of cephalopods (*Sepia officinalis*) it has been shown that through vitello vesicles, it is possible to transfer vitellin that will be used as a source of nutrients ^57^. A similar mechanism could be operating to transfer GSH to *O. maya* embryos, ensuring in this form that embryos can neutralize the ROS transferred from females. Previously, it has been observed in *O. mimus* and *O. maya* that although yolk consumption by embryos begins at the organogenesis stage, the highest rate of yolk consumption has been recorded in embryos in the activation stage onwards, suggesting during growth phase there are the highest energy demand^9,58^. Furthermore, in a detailed study of the embryonic development of *O. mimus*^17^ it was observed that the mobilization of reserves (glycogen, glucose, cholesterol, acyl glycerides, and proteins) from the yolk is significantly higher from the moment of activation of the circulatory system of the embryos until hatch, indicating that in the growth phase is when the embryo require higher quantity of metabolic energy. For this reason, it is possible to conclude that the most energetically costly stage is the activation stage, due to the synthesis of tissues, mobilization of nutrients, and consequently, the activation of organs and metabolic enzymes, all of these having repercussions on the respiratory metabolism and elimination of oxidative damage. In this sense, to answer the question, how hard is the octopus embryo’s life? The following hypotheses are put forward: (1) eggs were generated with relatively high levels of LPO and PO, indicating that a part of the maternal production of ROS during ovarian maturation was placed in the egg to be eliminated during embryo development (Fig. 6). ROS in *O. maya* eggs were controlled by the embryos during the growth phase of embryo. This was due to the activation of antioxidant defense mechanisms at stage XV, indicating the coupling between metabolic demands and the functioning of the antioxidant defense system against oxidative stress. This explains the increased expression of SOD and CAT enzymes during the organogenesis stage. Although the female transfers LPO and PO to the embryo in the yolk contents, the amount of GSH that is transferred to the embryos is sufficient to cope with them once the activity of GST, CAT, SOD enzymes is initiated. Preliminary expression analysis results during embryonic development (Publication in progress) indicate that in *O. maya* the glutathione system genes are overexpressed at the organogenesis stage indicating that, as with SOD and CAT, the glutathione system is in preparation for the neutralization of peroxidized lipids transferred from females. (2) When measuring HMR, stimulated by the increase in temperature (30 °C) embryos maintain a metabolic rate very similar to their RMR measured at 24 °C. This has important biological repercussions during development since, due to the multiple processes involved in organ formation and even the elimination of ROS transferred from the mother, this is reflected in a high metabolic rate even at their optimal developmental temperature. Therefore, even when stimulating a metabolic rate, it has a biological limitation caused by the processes involved in embryonic development. In this sense, an increase in temperature during embryonic development probably can modify the expression of these genes of the antioxidant system as well as other genes essential for proper embryonic development.

## Methods

### Embryos origin

*Octopus maya* embryos were obtained from nine (N = 9) wild mature females (400–700 g) captured on the continental shelf of the Yucatán peninsula, using the local drift-fishing method known as ‘Gareteo’. After being captured, animals were transferred to outdoor 6 m-diameter flow-through systems with shade mesh, protein skimmers, and 500 µm bag filters. Conditioning lasted 10 days (35 ± 1 salinity; dissolved oxygen (DO) > 5.5 mg L^*−*1^; 28 ± 1 °C) with a density of 1 animal m^*−*2^ and 2 PVC open tubes per animal as a refuge. Octopuses were fed twice daily (09:00 and 17:00) with a semi-moist paste made with squid and crab meat, gelatin, a vitamin-mineral premix, and ascorbic acid at a ratio of 8% of its body weight^59^. Also, four additional mature females were sampled after spawning to evaluate the antioxidant defense mechanisms (ANTIOX) and oxidant damage (OD) in the ovarian and unspawned eggs. The rest of the nine females were placed in 80 L individual tanks at 24 °C for 20 days and fed ad libitum with the same paste used during the conditioning period. A fiberglass box per tank was placed as a refuge and spawn settlement. Seawater in tanks was kept in semi-closed (5% water exchange d-1) seawater recirculation systems coupled with a rapid-rate sand filter. Water parameters showed similar values as above. pH was kept above 8 and photoperiod at 12L/12D with low light intensity (30 Lux m^*−*2^). The temperature of 24 °C was controlled with the air conditioning of the room. Spawned eggs from females were incubated at 24 °C for 55–60 days in 30 L seawater plastic tanks connected to a semi-closed recirculation system (two spawns per tank)^13^. The system was provided with mechanical, biological, and UV filtration. Water temperature was controlled with the room’s air conditioning, and parameters were kept similar to the female’s temperature treatment. Embryos were sampled every two days, covering all embryo developmental stages to the end of development: blastulation (stages 6–8; n = 310), organogenesis (stages 9–13; n = 396), activation (stages 14–16; n = 816), and growth (stages 17–19; n = 1165) (S-Fig. 1; Naef, 1928). Once sampled, the oxygen consumption of embryos was measured at 11 (n = 346), 24 (n = 912), and 30 °C (n = 356); After, embryos were weighed, photographed, immediately placed in liquid nitrogen, and stored at – 80 °C. Another group of embryos (n = 10 per stage from stage 6–19; 140 embryos in total) was photographed, separated from the yolk and chorion, and stored in RNA later until analysis.

### Oxygen consumption

Routine oxygen consumption was measured from the four stages, from blastulation to organogenesis stage, in embryos maintained at their incubation temperature of 24 °C, while thermal metabolic scope (a proxy of aerobic scope; Fry, 1947; 1971), of same embryo stages was obtained as the difference between the high and low metabolic rates. To do that, temperature was used to induce embryonic low and high metabolic rates using the temperature-induced metabolic rate or TIMR method^22^. With this method, it is possible to obtain standardized low (TIMR min) and high metabolic rates (TIMR-max) as a proxy of embryos standard^60^ and maximum metabolic rates, respectively. Pilot experiments conducted before the present work showed that a low metabolic rate (TIMR-min) of embryos could be induced when exposed to 11 °C (data not shown). TIMR-max was defined as the weight-specific oxygen consumption of an individual measured at 30 °C, the temperature at which the maximum metabolic rate of embryos was provoked^11^. Respiratory metabolism was measured in embryos individually placed in micro-plate clear glass vials with integrated sensor spots (1750 µL volume, Loligo Systems, Copenhagen, Denmark). Simultaneously, oxygen consumption of control chambers (vials without an embryo) was also measured. Vials were submerged in a transparent glass container with temperature-controlled seawater maintained at 11 or 30 °C. The container was placed on a Sensor Dish Reader (Loligo Systems, Denmark) that took oxygen concentration measurements every 15 s. The measurement time decreased with embryonic development from 40 to 20 min for TIMR-min and from 20 to 8 min for TIMR-max, depending of the developmental stage of embryos. All measurements were graphed according to time, and a linear 5–10 min segment of the curve was used to calculate the respiration rate:

$$M{O_2} = ({O_{2(A)}} - {O_{2(B)}})x\;(V/t)/M$$

where MO_2_ is respiration rate (mg O_2_ h^*−*1^ g WW^*−*1^), O_2 (*A*)_ is the initial oxygen concentration in the chamber (mg O_2_ L^*−*1^), O_2 (*B*)_ is the final oxygen concentration in the chamber (mg O_2_ L^*−*1^), V is the water volume in the chamber minus the volume of water displaced by the embryo, t is the time elapsed during measurement (h), and M is body mass of the embryo (mg WW).

Immediately after respiration rate measurements, eggs, and their corresponding embryos were photographed with a Leica EZH4D stereoscopic microscope equipped with a camera to identify the embryonic stage^30^, separated from the chorion, and weighed. The thermal metabolic scope (TMS) was defined as the difference between TIMRmax-TIMRmin while the factorial metabolic scope (FMS) was defined as mean TIMR-max/TIMR-min, resembling the factorial aerobic scope^61^. As this method lay on two distant temperatures, the Q_10_ temperature coefficient was calculated, as a complement, to give insights into the performance of the biological systems and the compensation capacity of the embryos: Q_10_ = (MO_2 *T*2_/MO_2 *T*1_)^(10*/T*2*−T*1)^.

### Antioxidant defense mechanisms (ANTIOX) and oxidative damage (OD)

The frozen embryos were individually homogenized in cold buffer 0.05M Tris pH 7.4 at 100 mg tissue/mL using a Potter Elvehjem homogenizer (Thomas Scientific, Pensilvania, US). Homogenate samples used for CAT, GST, and SOD were centrifuged at 10,000*g* for 5 min at 4 °C, and the supernatant was separated for analysis. All samples were stored at – 80 °C until analysis; all assays were duplicated. Catalase (CAT) activity was measured according to Góth (1991), modified by Hadwan and Abed^62^. In this method, undecomposed H_2_O_2_ is measured with ammonium molybdate after three minutes to produce a yellowish color with a maximum absorbance at 374 nm. Total glutathione (GSH) was measured with Sigma-Aldrich Glutathione Assay Kit (CS0260) (St. Louis, MO, US). This kit utilizes an enzymatic recycling method with glutathione reductase^63^. The GHS sulfhydryl group reacts with Ellman’s reagent and produces a yellow-colored compound read at 405 nm. The GST activity was determined from the reaction between reduced glutathione and 1-chloro-2.4-dinitrobenzene at 340 nm^64^. Proteins were analyzed in supernatant according to Bradford^65^ and used to normalize enzyme activities. SOD was evaluated using the Sigma-Aldrich assay kit (19160), which uses Dojindo’s (Molecular Technologies, Japan) highly water-soluble tetrazolium salt, WST-1 (2-(4-Iodophenyl)-3-(4-nitrophenyl)-5-(2,4-disulfophenyl)-2H-tetrazolium, monosodium salt) that produces a water-soluble formazan dye upon reduction with a superoxide anion. The reduction rate with O_2_ is linearly related to the xanthine oxidase (XO) activity and is inhibited by SOD. Therefore, the IC_50_ (50% inhibition activity of SOD or SOD-like materials) is determined calorimetrically. To evaluate oxidative damage (OD) caused by ROS, carbonyl groups in oxidized proteins (PO) were measured in the sampled embryos, estimating PO by using the 2,4-dinitrophenylhydrazine alkaline protocols developed by Mesquita et al. and reported in nmol/mg wet weight. For this assay, 200 µl of 2,4 dinitrophenylhydrazine (10 mM in 0.5 M HCL) were incubated with 200 µl of the sample homogenate and 100 µl of NaOH (6M). Absorbance was read at 450 nm after 10 min of incubation at room temperature against a blank where an equal volume of homogenization buffer substitutes the protein solution. Lipid peroxidation (LPO) was evaluated using Peroxi-Detect Kit (PD1, Sigma-Aldrich, USA) following the manufacturer’s instructions. The procedure is based on the fact that peroxides oxide iron at acidic pH, Fe^3+^ ion will form a colored adduct with xylenol orange measured at 560 nm. To quantify lipid peroxides only, an extra set of samples were treated with 10 µl 10 mM of triphenylphosphine^66^.

### Gene expression during the embryonic development

#### Biological material

Embryonic stages were divided into four major groups: (1) Blastulation (includes stages 6–8 (n = 30) represents the first cellular divisions until the formation of the blastula; (2) Organogenesis (characterized by tissue differentiation into organs) defines the stages 9–13 (n = 50); (3) Activation (circulatory and respiratory systems) starts to function from stages 14–16; n = 30); and (4) Growth (includes stages 17–19, all organs are entirely differentiated, and the circulatory and respiratory systems are completely functional (n = 30).

#### RNA extraction and first-strand cDNA synthesis

Total RNA of the complete embryo was extracted with TRI Reagent (SIGMA) following the manufacturer’s instructions. RNA was quantified with a NanoDrop 2000 Spectrophotometer (Thermo Scientific, DE, USA), and the integrity was evaluated by 1.0% agarose gel electrophoresis using 1× TAE buffer. Residual DNA was digested with DNAse I (RQ1 DNase, Promega, WI, USA) at 1 U per µg of RNA and purified by adding 0.1 volumes of sodium acetate 3M and three volumes of absolute ethanol. Effective DNAse treatment was confirmed by verifying no-amplification after PCR using Elongation factor 1-beta (EF1*β*) as control. PCR amplification was performed in a 15 µl reaction volume that contained 1× colorless buffer, four mM of MgCl2, 0.2 µM of dNTP mix, 0.2 µM of each primer (Table 1), 1U GoTaq Flexi Polymerase (Promega, WI, USA) and 1 µl of clean RNA as a template. PCR consisted of 40 cycles, denaturing at 94 °C for 30 s, annealing at 60 °C for 30 s, and extension at 72 °C for 15 s. PCR products were visualized on agarose gel (2%). Complementary DNA (cDNA) was synthesized from 1 µg of clean RNA in a final volume of 20 µl using ImProm-II TM reverse transcriptase (Promega, WI, USA) following the manufacturer’s instructions.

**Table 1:** Nucleotide sequences of primers used in qPCR analysis.

Gene	Primer name	Seq. 5′–3′	T.A. (°C)	Size (bp)	Amplific. Effi. (%)
CAT	Om_CATA-F Om_CATA-R	TCCGTCCCTTTGATAGTTGGC CAGGAATTGAACCAAGCCCG	60	140	82.3
CAT	Om_CATA-F Om_CATA-R	TCCGTCCCTTTGATAGTTGGC CAGGAATTGAACCAAGCCCG	60	140	82.3
MnSOD	Om_SOD-Mn-F Om_SOD-Cu-R	AATGGTGGAGGCCATCTCAA TCCAACGATGCTGTTTGGTC	61	182	106.6
HIF1A	Om_HIF1-F Om_HIF1-R	AAAGGACAGATCACCACGGG AGAGTGGTTTCTGAGTGCGG	60	112	90.5
EF-1-beta	Om_Ef1b-F Om_Ef1b-R	TGATGTCAAACCATGGGACG AGAGGTGCTAACTTGGACGC	60	108	99.1
Gpam	Om_Gpam-F Om_Gpam-R	ATAACGCCAAAGAGAGAGCACA CCGGACATCATTTTCCCACAAC	60	179	90.8
hnRNP D-like	Om_Hnr-F Om_Hnr-R	GTTCTCGTGGATTTGCTCGC TCCAGAGGTTTTGGTTTTGTCC	60	121	97.4
TufM	Om_TufM-F Om_TufM-R	TTTTCCCCATAGATTCGGCTGT AAGCACCGTGTCCCAGTATATC	60	117	88.5

#### qPCR analysis

The amplification efficiency for each primer was estimated by standard curves obtained from serial dilutions (dilution factor 1:5) of a cDNA pool from all samples. Four potential reference genes previously validated in *O. maya*^67,68^: Elongation factor 1-beta (EF1*β*), Heterogeneous nuclear ribonucleoprotein D (hnRNP), Elongation factor TU, mitochondrial (TuFM), and Glycerol-3-phosphate acyltransferase 1, mitochondrial (Gpam) were included. Efficiency for reference and target genes was estimated from each standard curve using the equation: E = (− 1 + 10^(*−*1*/slope*)^)^69^. The qPCR was conducted triplicate on a CFX96 Real-Time PCR Detection System (Bio-Rad). The qPCR reactions were conducted following the protocol described by Ventura et al.^68^, using primer-specific annealing temperatures (Table 1). The stability of reference genes was evaluated using RefFinder^70^. Relative expression of the target genes was estimated from relative quantities as Hellemans et al.^71^ proposed. Logarithmic transformation was carried out for all relative expression values. Analyses were performed using STATISTICA 8.0 (StatSoft, Tulsa, OK, USA), with a statistical significance of P < 0.05. Values were plotted in their original scale. No standard errors bars are presented in the graphs because, differently from the means, the variances of transformed variables are not back-transformable^72^.

### Ethical statements

1. All experimental protocols were approved by a named institutional and/or licensing committee. This project was approved by Comisión de Ética Académica y Responsabilidad Científica, of Universidad Nacional Autónoma de Mexico CEARC/Bioética/25102021.
2. All methods were carried out in accordance with relevant guidelines and regulations, which were taken from the European Commission regulation (2010/63/EU).
3. Although there is no regulation related with the work with Octopus embryos we follow the arrive guides in attempt to obtain enough information of this key phase of the life cycles of cephalopods.

### Supplementary Information

Supplementary Information.

## Data Availability

Data are available at: 10.5281/zenodo.11360890.
