# Supplementary material for: The hard life of an octopus embryo is seen through gene expression, energy metabolism, and its ability to neutralize radical oxygen species
Source: Sci Rep. 2024 Jul 17;14:16510. doi: 10.1038/s41598-024-67335-9 (PMC11255218; doi:10.1038/s41598-024-67335-9)

S1. Octopus maya embryo through development: blastulation (A), Organogenesis (B), Activation (C) and growth (D)


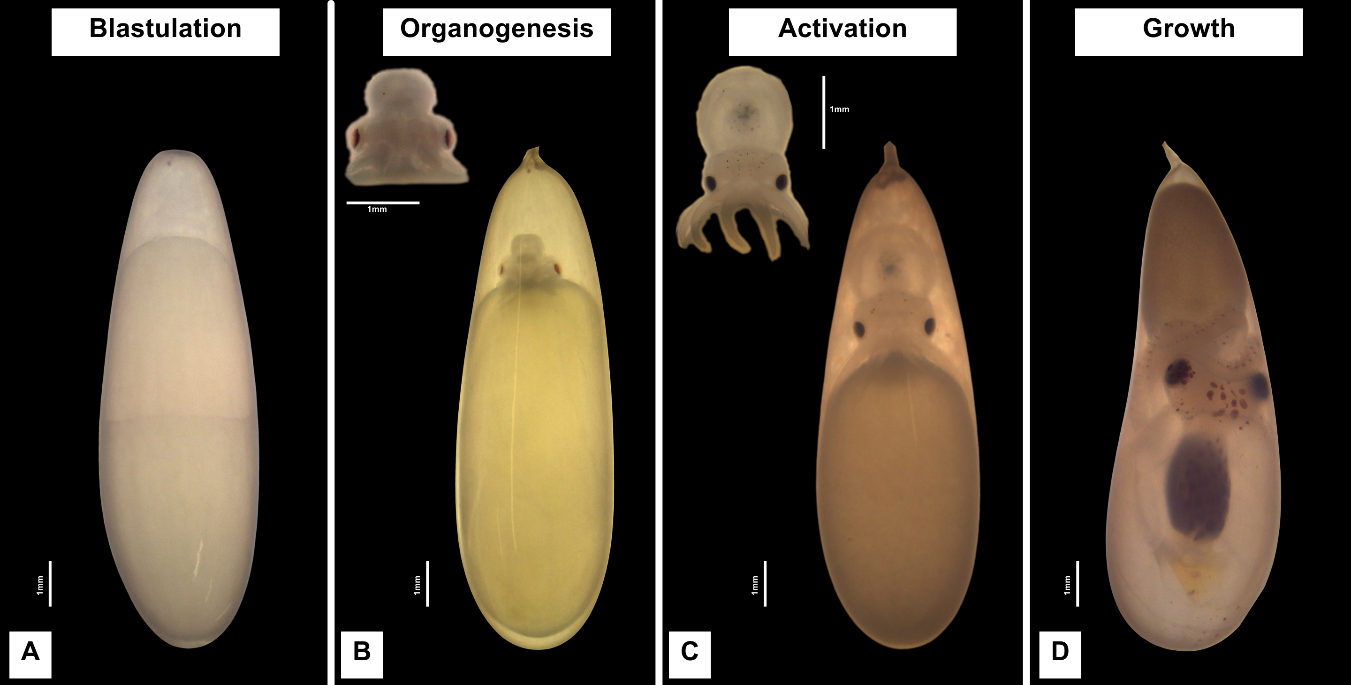

Supplement: Supplementary file 1 — Supplementary Information. [file 41598_2024_67335_MOESM1_ESM.docx]
